# Restructuring breeding programs 1: Integration of diversity

**DOI:** 10.1186/s12711-026-01046-9

**Published:** 2026-04-28

**Authors:** Tobias A. M. Niehoff, Jan ten Napel, Mario P. L. Calus

**Affiliations:** Animal Breeding and Genomics, Wageningen University & Research, Droevendaalsesteeg 1, P.O. Box 338, 6700AH Wageningen, the Netherlands

## Abstract

**Background:**

Closed populations are always at risk of losing genetic diversity needed for long-term genetic progress. When selecting, breeders are sometimes afraid of “missing out” on rare and/or beneficial genetic variation. Such genetic variation missing in a high-performing nucleus population may be found in low performing animals. The gap in performance level may hamper utilization of external genetic variation in the nucleus. In this study, we test different indicator variables to select animals as donors of diversity to be added to a commercial nucleus population via simulations. We also test a strategy of integration of external diversity found in those donors into an elite population in an ongoing genomic selection breeding program that employs optimum contribution selection. The core idea of restructuring breeding programs is to create layers through which diversity is gradually “upcycled” until it is ready for integration into the elite population. This strategy is assessed by means of simulation of a purebred pig breeding program over a time horizon of 20 generations. We screened boars as diversity donor candidates that have been selected in previous generations.

**Results:**

The introduced variation did not help to outperform the genetic progress achieved by the control scenario (no diversity introduction) in any of the tested scenarios, though some scenarios did not perform much worse. Generally, the more resources are spent on the upcycling-component, the more the diversity in the elite can be increased, but this compromises genetic gain because less resources are available to advance the elite. Although the effects on genetic gain and diversity were similar among the diversity-selection-criteria, selecting boars with the lowest kinship to the elite population was slightly more effective and is the easiest strategy to implement, relative to selecting boars with unique haplotypes or haplotypes with a higher breeding value than in the elite. The efficiency of turning genetic diversity into genetic gain was always enhanced in breeding programs investing in diversity introduction.

**Conclusions:**

Though the tested layered breeding programs did not show an advantage in terms of gain over the control scenario, the strategies may be relevant for real breeding programs (1) if a population is lacking diversity for a trait or (2) if new traits become important.

**Supplementary Information:**

The online version contains supplementary material available at 10.1186/s12711-026-01046-9.

## Background

Genetic variation is crucial for genetic progress in breeding programs. Without mutations creating new variation, the genetic diversity of a population of finite size will decrease over time due to drift. The FAO suggests for animal populations to target an effective population size (Ne) of at least 50 to guarantee survival of the population in the short and medium term [1, p. 141]. Breeding organizations see the need for and commit to breeding responsibly, of which maintaining genetic diversity is a part, as can be seen for example in the list of the 33 organizations that have voluntarily adopted the Code-EFABAR for responsible breeding [2].

The recommendation of keeping an Ne of 50 translates to a maximum expected increase in inbreeding level per generation (long-term inbreeding rate ($$\varDelta F$$)) of 1% per generation as can be derived from $$\varDelta F=1/\left(2Ne\right)$$ [3]. To understand the impact of the effective population size, one can use the Ne to calculate the average inbreeding level in a future generation as $$1-{(1-1/(2Ne\left)\right)}^{n \; generations}$$, or calculate the generation in which a certain average inbreeding level is to be observed as log(1−F)/log(1−(1/(2Ne))). For single loci, e.g., the Ne can be used to calculate the probability to keep an allele in the population for a certain number of generations given the allele’s starting frequency $$\left({p}_{i}\right)$$ $$\left( {P\left( {keep\;allele\;i\;for\;n\;generations} \right)} \right. = $$ $$ \left( {1 - P\left( {loose\;allele\;i\;} \right.} \right. $$$$\left. {\left. {\;in\;next\;generation} \right)} \right)^{{n\;generations}} $$$$= {{{\left( {1 - {{\left( {1 - {p_i}} \right)}^{2Ne}}} \right)}^{n\;generations}}}$$ [4]. For instance, there is a 10% probability not to lose alleles at 45 independent loci, all with an initial frequency of 5%, for 10 generations. These equations illustrate applications that help to choose a desired Ne. They also illustrate that even for populations with an Ne of 50, which is observed in some commercial populations [5–7], variation will be lost sooner or later.  

Despite all efforts to balance genetic gain and diversity, the inbreeding level of closed populations rises, as reported for dairy cattle for example [5, 8–10]. In turn and also caused by the effects of selection, genetic variances and thus long-term genetic gain may decrease over time, which has been shown in pigs [11], broilers [12], dairy sheep [13], and dairy cattle [14]. It should be noted that the impact and extent of inbreeding and loss of variance varies among species and breeds, and is not always critical.

While selection reduces the variance for selected traits, inbreeding reduces the genetic variance for all traits regardless of whether they are under selection or not – at least in theory. Thus, the genetic variances of traits that were not targeted by selection until around the year 2000, but have become more relevant thereafter, such as health [15], welfare, or environmental-sustainability traits [15–20], might have been reduced due to past inbreeding without having been selected on [21].

Genetic variation could be increased by the importation of genetic material from other breeds or from material stored in a gene bank, e.g., cryopreserved semen. Cryopreserved germplasm of many important livestock species is already stored in gene banks [22–24]. However, only a handful of studies report on the use of gene bank material to restore genetic diversity [25–27], which might indicate limited interest in the preserved genetic material, or a lack of strategies to integrate genetic material from unrelated but potentially lower performing animals into a breeding population under ongoing selection. Studies by Doekes et al. [28] for Holstein and by Eynard et al. [29] for the Meuse-Rhine-Issel Dutch cattle breed both showed that more genetic gain in the total merit selection index is possible while maintaining the same level of average relatedness in the population by using optimum contribution selection (OCS) [30] and by including old bulls with cryopreserved semen in the gene bank. However, Doekes et al. [28] note that the impact may be limited without changes in the selection index with respect to the weights of the traits, which was also shown in [31, 32]. Using simulations, Leroy et al. [31] showed that using semen from cryobank bulls in a population under selection has no lasting effect on gain and diversity unless the selection index is changed, i.e., a historically important trait becomes less important (see their Fig. 2). A crucial aspect that has not been addressed by the mentioned studies is how to handle offspring stemming from cryopreserved bulls in the next generation. Those offspring are lower performing, which makes them uninteresting for farmers, but their low relatedness to the population makes them, or rather their descendants, interesting for future breeding. No special selection strategy was implemented for such descendants in Leroy et al. [31], which is likely why they are not selected based on their index value in subsequent generations.

In this study, we refer to external animals that are selected based on their “uniqueness” to contribute to the population as “diversity donors”, following Sanchez et al. [33]. Diversity donors are typically lower performing with respect to the selection index than animals considered to be “elite”. The offspring of a donor x elite mating are unlikely to be selected because of low performance and the to-be-introduced diversity may be lost after one generation, as explained before. To prevent that, the diversity must be introduced into a higher performing genetic background first. We call this process “upcycling” in this study. This has been studied in the field of plant breeding, where it is called “pre breeding” [34, 35] or “bridging” [36, 37]. In its core, it can be seen as backcrossing with some tweaks added to it. Upcycling has two purposes: in addition to improving the low performance level associated with the introduced diversity, upcycling also serves to test out and learn about the diversity. This testing is important, as diversity that seemed promising may turn out to be not as good once it is integrated into a different genetic background. Once the descendants of diversity donors are on a comparable performance level as the elite, they may be included in the elite population.

We propose to include the upcycling component by adding several layers in the breeding program. The upcycling component requires some resources in terms of matings or barn space, which implies that less resources are available for the elite population. This reduction in elite population size will cause a reduction in genetic gain, at least in the short-term until the new diversity enters the elite population. This temporary reduction would either have to be accepted or countermeasures need to be taken, e.g., by temporarily increasing selection intensity. 

The objectives of this study were (1) to test if breeding programs can be restructured to enable introducing missing diversity for quantitative traits by adding non-elite animals to the breeding program with the proposed upcycling scheme, and (2) to test different indicators of diversity for selection of non-elite animals to be introduced. We evaluate these strategies by means of simulation of a simplified purebred swine breeding program. As a straightforward example, the diversity introduction scenarios considered are based on using animals of the same line from previous generations, of which frozen semen samples are stored. Selection in all scenarios is done with OCS as the current gold standard for diversity management. Thus, we compare our strategies to the optimal breeding practice.

## Methods

### Upcycling breeding program

**Fig. 1:**
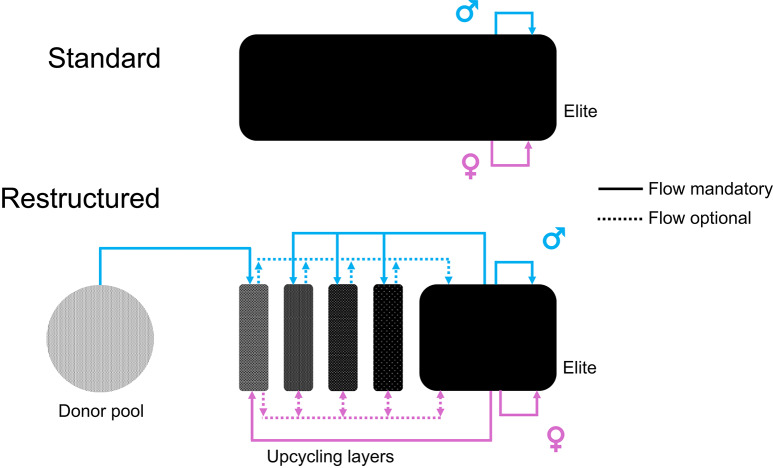
Schematic representation of the standard and restructured breeding programs. Shown are a standard breeding program without upcycling layers and a restructured program with upcycling layers for diversity introduction. Colors indicate if a male (blue) or female (pink) individual is used

**Fig. 2:**
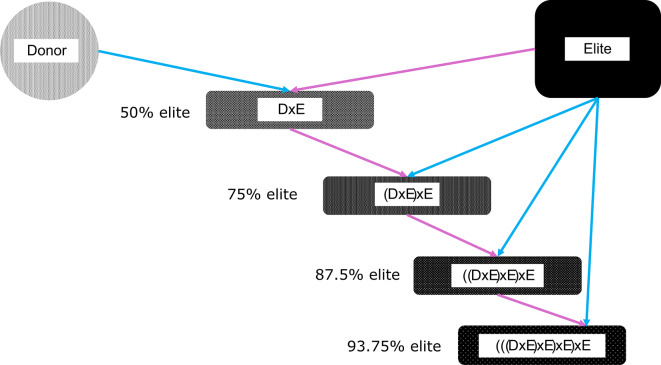
Backcrossing scheme and expected percentage of elite genome contribution. Colors indicate if a male (blue) or female (pink) individual is used

The structure of the envisioned upcycling breeding scheme is shown in Fig. 1 and was simulated in this study. To accommodate the layers, the size of the elite population is reduced. Boars selected as diversity donors are crossed with elite sows that are not selected for population improvement. Their F1 offspring enter the first layer. The descendants of the donor x elite-matings may then be selected to serve as parents in the following layers or in the elite population, depending on their estimated breeding value (EBV) and their genetic rarity. We envision such a restructured breeding program scheme to allow selection of animals from and to all components. Only allowing directed flow between adjacent components (e.g. F1 in layer 1, their offspring must go to layer 2, their offspring to layer 3, and so forth) would not allow interesting genetics occurring in the elite population to be picked up in the upcycling scheme, or would not allow difficult-to-extract-variation to stay in the upcycling scheme longer, or would not allow high merit F1 offspring to be included in the elite population. However, in its simplest form the layer-component can indeed be understood as a backcrossing scheme (Fig. 2) with individuals born into layer 4 having 93.75% alleles derived from the elite.

### Identifying diversity

Many different indicators of diversity exist. We propose to categorize these indicators into “exploitation” and “exploration” diversity indicators. Diversity indicators for exploitation assign a value to the diversity. A locus with a known beneficial effect, i.e., a major QTL, that is not segregating in the elite population is an example of diversity that can be exploited (e.g. introgression of the SLICK1 allele from Senepol into Holstein cattle for improved thermotolerance [38]). In contrast to exploitation, diversity that is described by exploration indicators does not have an immediate known value. Diversity donors carrying exploration-diversity must first be brought into the population. Their descendants can then be used to evaluate if the donor carried alleles that are valuable to the breeding objective. An example of exploration diversity is a low kinship to the elite population, as a diversity donor with low kinship to the elite population may carry many rare or even missing alleles whose effects are not known.

**Fig. 3:**
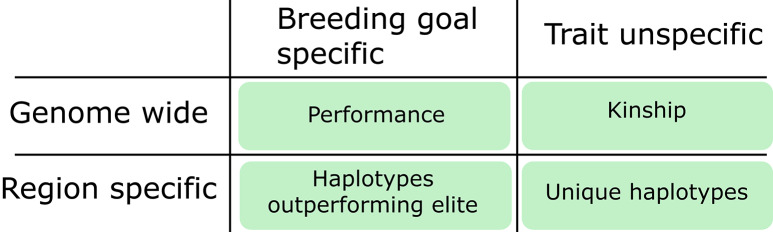
Classification of diversity metrics. Categories to classify diversity metrics and the metrics we used in this study (in green)

Not only can diversity metrics be categorized into “exploitation” and “exploration”, but also for describing genome-wide diversity and locus-, or region-specific diversity. The major-QTL-example falls into the category of region-specific diversity, whereas the average kinship of an animal to a population describes its genome-wide diversity. The four resulting categories are shown in Fig. 3. In this study, we consider the estimated breeding value of an animal to reflect its selection index, meaning that it is a genome-wide indicator for useful genetics with respect to the breeding goal.

Following Allier et al. [39], we use the estimated breeding value of a haplotype as a criterion that applies to a specific region in the genome and is meant to be used for exploitation. It describes the value of a chromosome segment and can be used to investigate if diversity donors carry haplotypes that outperform haplotypes at the same position in the elite. Its exploration-oriented counterpart is a metric we call “unique haplotypes”. The difference is that the latter describes whether a haplotype found in the pool of potential donors is missing from the elite population without knowing the effect of the haplotype. The aforementioned low kinship to elite animals was used as a genome-wide and exploration-oriented indicator of diversity.

The following three sections explain the diversity metrics (Fig. 3) that were used to select donors in this study. Short R scripts for testing are provided in Additional File 1 Code S1, Additional File 2 Code S2 and Additional File 3 Code S3.

### Diversity metrics

#### Rare beneficial haplotypes

A method to select donors that carry rare beneficial haplotypes was proposed by Allier et al. [39] as an extension of the optimum haploid value (OHV) [40], and that method aims to stack up as many beneficial haplotypes as possible in a population. The OHV was proposed as a mate selection criterion and thus only considers the haplotypes of two individuals. Similarly, the value of a group is the hypothetical breeding value that would be observed if all the best haplotypes were fixed. Allier et al. [39] named it the “H-criterion” but we will use the term “rare beneficial haplotypes” here. This concept, in varying forms, has been proposed several times under different names, such as “optimum population value” [41], “haplotype stacking” [42], “genotype building” [43], or “selection limits” [14, 44]. Note that “selection limit” is not a process but a value that indicates the theoretically highest attainable genetic value if all beneficial alleles were fixed in the population. The process of selection to keep the selection limit as high as possible is conceptually identical to the other mentioned strategies.

From a maximization point of view, selecting animals with the highest EBV aims to maximize the population average breeding value in the next generation. The time horizon for combining good haplotypes on the other hand is not defined, but is generally long-term. The only influence one has on the time horizon is in the choice of the haplotype length: the shorter the haplotypes, the more recombinations, or generations, are needed to combine them all in one genome.

In this study, we follow the selection procedure based on rare beneficial haplotypes, as proposed in Eq. 5 of Allier et al. [39] and illustrated in Additional File 4 Fig. S1. First, a set of elite animals is selected. Next, the chromosomes of elite animals and available donors are split into segments and the estimated breeding value of each segment is calculated by multiplying the estimated SNP effects with the genotype states. Then, the estimated breeding values of the best haplotypes of every segment of the elite are summed, followed by selecting the donor whose haplotypes would increase the maximum possible estimated breeding value the most. This is of course only possible if the donor carries at least one haplotype with a higher estimated breeding value than any other elite animal does at the same position in the genome. If a second donor should be selected, the previous selected donor is added to the group of elite animals and the procedure starts again. This is to avoid selecting a second donor that has the same outperforming chromosome segments as the first selected donor.

In this study, we used code from Supplementary file 2 of Allier et al. [39] for the calculation of haplotype estimated breeding values and the selection based on them. Allier et al. [39] used segment sizes of 100 consecutive SNPs moving along the chromosome in increments of 20 SNPs. Given the number of maize chromosomes of 10, the average chromosome length of 1.9 Morgan and the 40k SNP panel, their average segment size is about 5 centimorgan (cM) long. In our study, we used 44 SNPs per segment to target an average length of 4 cM. The segments moved along the chromosome in increments of 20% of the segment length. An example R script for our implementation is provided in Additional File 1 Supplementary Code S1.

**Fig. 4:**
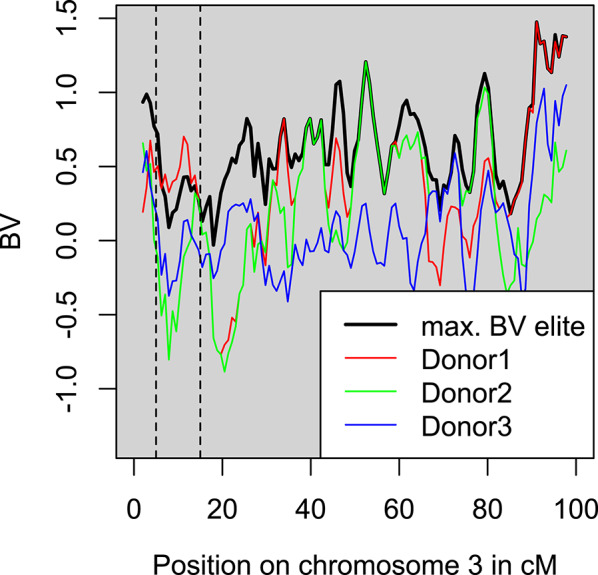
Example of the highest haplotype-breeding value along a chromosome. The highest breeding value of a haplotype per position in the elite population without donor introductions and the breeding value of the best of the two haplotypes of three selected donors (colored lines). Dashed lines indicate the region in which Donor1 carries haplotypes with higher estimated breeding value than any elite animal. This figure was made based on a small simulated data set

Figure 4 visualizes the detection of good segments in a chromosome in a small simulated data set. At most positions, the best haplotype is carried by one of the elite animals. However, at around position 10 cM, donor 1 (in red) carries a haplotype with a higher estimated value than any other haplotype in the elite. The figure also shows that donors carry haplotypes with lower EBV at most other positions.

#### Rare haplotypes

An issue with using estimated SNP effects for selection of unrelated animals is that alleles that may segregate in the pool of potential diversity donors may not be present or have a different frequency in the population that is used to train the prediction model. An allele that is fixed in the elite population will thus get an effect size of zero, and the effect of the haplotype will be estimated incorrectly. Also, estimated SNP effects only indicate the effect of QTL they are in linkage disequilibrium with, and the effect of a QTL is distributed among the estimated effect sizes of several neighboring SNPs. Thus, indirect predictions, i.e., predictions only based on estimated SNP effects without own or offspring phenotype records, are only meaningful if the linkage disequilibrium (LD) of the genomic prediction training set is somewhat similar to the LD of the test set [45, 46]. Thus, it is to be expected that the breeding values, and thus also the haplotypes, of animals that are more detached from the training population are predicted with lower accuracy [47–50]. In addition, breeding values for segments are not perfectly estimated, even for animals with a high relationship to the training population, simply because the prediction accuracy of EBVs is never perfect.

Selecting to maximize the summed EBV of the best haplotypes means selecting a group of individuals that carry the estimated best possible haplotype at every position in the genome regardless of the frequency of that haplotype. An alternative strategy that does not rely on effect-estimates is to increase the number of unique haplotypes at every position in the genome. Assuming that every haplotype has a different but unknown breeding value, simply increasing the number of unique haplotypes should maximize the chances that one of them is the best one. Thus, increasing the number of unique haplotypes should increase the maximum achievable breeding value, i.e., the selection limit, as well.

This could also be done for SNP alleles, i.e., a haplotype consisting of just one locus. However, alleles of loci on the SNP chip are unlikely to be missing because loci included on the SNP chip are usually selected to have segregating alleles in the population with a high minor allele frequency. Thus, it is less likely that alleles at genotyped loci will be missing compared to alleles at ungenotyped loci with lower minor allele frequencies. If no locus on the SNP chip is fixed, no diversity introduction seems necessary, as all genotyped alleles are present in the elite population. However, breeding programs do not work with complete sequence data and, thus, the fixation-status at ungenotyped loci is unknown. Yet, variability at loci that are not on the SNP chip is also of interest for breeders. The choice to work with haplotypes is motivated by the assumption that a unique combination of genotyped SNP alleles also carries a unique combination of QTLs (at ungenotyped loci) that may be missing in the elite population.

**Fig. 5:**
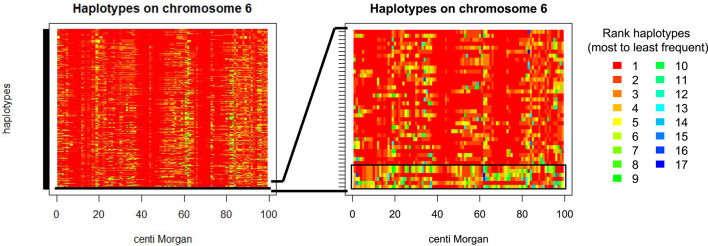
Visualization of haplotype diversity in the elite population and three donors. The donor haplotypes are within the black rectangle. Data of a random replicate was used. One chromosome is plotted. Segments are 1 cM long. Haplotypes are colored according to the rarity and number of unique segments with red being the most common haplotype and deep blue being the 17th (least) common haplotype. Some positions (columns) do not show any blue because less than 17 unique segments are present. Haplotypes are ordered with segments carrying common haplotypes on top

The number of unique haplotypes was calculated for fixed 1 cM chromosome segments. The exact procedure is described in the Additional File 4 Fig. S2. Animals that increase the total number of unique haplotypes in the population the most were selected as donors, similar to the procedure used for selecting based on rare beneficial haplotypes. A visualization of selecting for many segregating haplotypes is in Fig. 5, which shows that selected diversity donors carry some haplotypes that are not present in the elite population. Similar to selection for rare beneficial haplotypes, selection for unique haplotypes ignores the genetic background of the animal, i.e., linkage to other haplotypes and the performance level.

In this study, we used haplotypes of 1 cM length without overlap between consecutive segments. Example code for the calculation and selection of unique haplotypes is provided in an R script in Additional File 1 Supplementary Code S2.

#### Low average kinship to the elite

Selecting donors based on their kinship to the elite population is probably the most straightforward method of identifying diversity. In this study, a kinship matrix was constructed based on shared identical chromosome segments based on genotyped SNPs with the function “segIBD()” of the optiSel R package [51]. In order to be considered identical, the minimum length of a shared segment was set to 4 cM and to have at least 20 consecutive identical SNPs. Animals that showed the lowest average kinship to current elite animals were selected as donors.

The decision to calculate kinship based on segments of identical haplotypes is based on preliminary comparisons of the true kinship to different kinship estimators. The segment-based kinship measure was identified as the most accurate one among estimators that do not require assumptions about the base population. Our decision process is explained in the Appendix.

### Selection

#### Donor selection

**Fig. 6:**
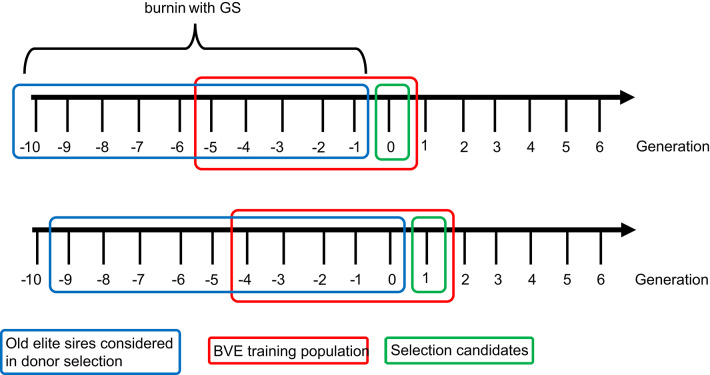
Generation of selection candidates, animals in the breeding value estimation (BVE) training population and old elite sires considered as potential donors. Example shown in generation 0 and 1 of the breeding program

In this study, all boars that were selected as elite sires in the past 10 generations are considered for selection as diversity donors (Fig. 6). We assumed that some semen samples of these animals are readily available, for example in an in-house cryo tank. The diversity donors were selected after selection of the elite animals and after dams were selected for the layer-component. This is to avoid selecting boars whose alleles are already present in animals that get to contribute to the next generation. 

#### Selection in the upcycling-component

An overview of the possible flow of animals is given in Table 1. Sows to be used as parents for layer 1 come from the elite population and are not used to advance the elite population (Fig. 1). Sows for layers 2 and 3 are selected based on one of the diversity metrics described before, whereas sows for layer 4 are selected based on their EBV (in this study a genomic EBV (GEBV)). Sows of all layers are inseminated with semen of the best elite boars selected for elite population improvement. Sows for layer 2 may be selected from any layer and the elite population. This also applies to sows for layer 3, except that animals born in layer 1 are ignored to avoid that always the most diverse but least performant animals are selected. Sows to be used in layer 4 are selected based on their GEBV to increase the chance of their offspring to reach the elite performance level. Sows for layer 4 were only selected from the pool of animals born in the upcycling component to avoid selecting high GEBV elite animals. In this study, semen of a diversity donor was used to inseminate one sow, i.e., the number of sows used in each layer was identical to the number of diversity donors. Sows for the upcycling component were selected after sows for the elite were selected based on their GEBV. Upcycling is done via females and males born in the upcycling layers are generally discarded, as they would require more housing resources without being used much. The only chance for a male born in the upcycling component to be used is by selection as an elite sire by OCS (Fig. 1).

**Table 1:** Overview of number and origin of selected animals in the 13 selection strategies

Selection strategy	Donors	Sows to produce layer 1	Sows to produce layer 2	Sows to produce layer 3	Sows to produce layer 4	Sows to produce elite	Sires to produce elite
1/2/3 (rare beneficial haplotypes)	3/10/25	3/10/25	3/10/25	3/10/25	3/10/25	388/360/300	Variable number (OCS)
4/5/6 (rare haplotypes)	3/10/25	3/10/25	3/10/25	3/10/25	3/10/25	388/360/300	Variable number (OCS)
7/8/9 (low kinship)	3/10/25	3/10/25	3/10/25	3/10/25	3/10/25	388/360/300	Variable number (OCS)
10 (low kinship)	10	10	10	10 (EBV from layer 1–2)	–	370	Variable number (OCS)
11 (low kinship)	10	10	10 (EBV from layer 1)	–	–	380	Variable number (OCS)
12	8 (low kinship) 4 (rare haplotypes) 5 (rare beneficial haplotypes, selected from past 5 generations)	8 4 5	8 (low kinship) 4 (rare haplotypes) 5 (rare beneficial haplotypes)	5 (EBV from layer 1–2)	–	361	Variable number (OCS)
13 (control)	–	–	–	–	–	400	Variable number (OCS)
Order of selection:	7	3	6	5	4	1	2
Selected from (if not stated otherwise):	Selected sires for elite of past 10 generations based on diversity criterion	Best females according to EBV from layer 1–4 or elite	Best females according to criterion born into layer 1–4 or elite	Best females according to criterion born into layer 2–4 or elite	Best females according to EBV born into layer 1–3	Best females according to EBV born into layer 1–4 or elite	All males from layer 1–4 and elite; selected by OCS (maximize gain while constraining inbreeding rate to 1%)

**“**Order of selection” indicates the chronological order in which groups of animals were selected as some selection decisions are influenced by the choice of previously selected animals. Column “selection strategy” indicates the ith selection strategy. For example, “1/2/3” means these are the first, second and third strategies tested. They only differ in the proportion of resources used for upcycling and are combined in one row for brevity

We tested the restructured breeding program with three sizes of the upcycling component (3, 10, and 25% of the overall breeding program) for the three diversity criteria rare beneficial haplotypes, rare haplotypes, and low kinship. In addition, we tested three selection strategies that were designed after evaluating some initial results. Their aim was to reduce the upcycling component while trying to retain the benefits that scenarios with more emphasis on upcycling had. In the first two schemes, 10 donors were selected based on low average kinship and only 3 or 2 layers were used for upcycling. Consequently, only 30 (7.5%) or 20 (5%) sows were needed in the upcycling component. The third scheme was a combination of all criteria, in order to bring in exploration-diversity, and once some information was generated for this diversity-type, exploit it. In this scheme, 8 donors were selected from up to the last 10 generations based on low average kinship and 4 donors based on carrying missing haplotypes. Selection with these metrics was done for two layers. In addition, 5 donors from the past 5 generations were selected based on carrying rare beneficial haplotypes. Selection based on rare beneficial haplotypes was allowed for 3 layers. After the diverse genetics were explored and partly crossed into the elite genetic background, the idea was that useful haplotypes can be identified and then exploited by selection based on rare beneficial haplotypes. This selection scheme requires 9.75% of all sows for the upcycling component and was implemented so that all available sows could be selected by every criterion. This means that a sow can be selected by any criterion, regardless of the criterion that her mother or any other ancestor was selected on.

#### Elite selection

For the elite component, females were selected based on their GEBV (Table 1). Sires were selected and contributed to the next generation by the amount determined by OCS [30] using the R package “optiSel” [51]. All males born in the elite component and the upcycling component were candidates in OCS, but diversity donors were not. A restriction on the maximum contribution corresponding to 30 semen doses per boar was set to mimic biological limitations but this limit was hardly ever reached. In addition, we imposed a restriction that the minimum contribution of a boar should either be 0 or correspond to at least 1 mating to avoid selecting boars with very low contributions. This was implemented by running optiSel iteratively and setting the contribution of the boar with the lowest contribution to 0 if its determined contribution was not higher than at least 1 mating. Elite sires could be selected from the elite or the upcycling components of the breeding program (Table 1).

The contributions were optimized to maximize genetic gain while limiting the increase in average kinship to at most 1% per generation to achieve an Ne of at least 50. The maximum kinship level of the next generation was calculated as ${\stackrel{-}{f}}_{t+1}={\stackrel{-}{f}}_{t}+\left(1-{\stackrel{-}{f}}_{t}\right)*0.01$, with ${\stackrel{-}{f}}_{t}$ and ${\stackrel{-}{f}}_{t+1}$ representing the average kinship in generations *t* and *t*+1. We used a genomic relationship matrix based on method 1 of VanRaden [52] to infer kinship. As reference allele frequencies, i.e., the frequencies used to center allele counts of individuals with the P-matrix in the GRM creation, we used the allele frequencies observed in the elite population 5 generations before the current generation. This decision is based on findings of Gautason et al. [53], who reported the highest genetic gain per unit kinship increase when the allele frequencies of the base generation of old sires are used. We chose this hybrid solution of a “moving” but still somewhat old base generation because inferring base allele frequencies may not always be possible, to allow some movement of allele frequencies due to selection, and because our preliminary investigation showed high within-generation correlation to true identity-by-descent (IBD) kinship when using allele frequencies of 5 generations ago (Appendix, Fig. 11b) for centering. True IBD is measured by the proportion of shared founder genome content by tracing back chromosome segments to founders. This is calculated with function “kinship.emp()” in the simulation software MoBPS [54].

### Breeding program simulation

The simulation of the pig breeding program of [55] was adopted, using the R package MoBPS (version 1.11.59) [54]. In brief, 6000 purely additive QTL were placed randomly on the 18 chromosomes. All animals were genotyped with a 20,000 SNP array which did not include positions of the QTL. After population history simulation as in [55], SNPs for genotyping were selected based on high minor allele frequency among all available SNPs in the genome (about 65k), as is common practice in SNP chip design [56, 57]. For more details, we refer the reader to [55]. All animals were phenotyped for daily gain (heritability = 0.3 in the base generation). Every sow was inseminated with semen of a single boar. A total of 400 sows were selected every generation. Litter size was set to 6. Thus, the population size was 2400 per generation.

Ten generations of burnin were simulated with truncation selection based on genomic estimated breeding values (GEBV). During burnin, 40 males (400 females) were selected out of 1200 (1200) candidates. These numbers varied after the burnin phase for the male side due to selection with OCS. Mating between males and females was always at random. The total population size was kept constant but with varying sizes of the upcycling component by reducing the number of selected elite females. 20 replicates of each scenario were simulated.

A genomic relationship matrix based on method 1 of VanRaden [52] was used for breeding value estimation. The training population for breeding value estimation was comprised of animals in the current and the previous 5 generations. All animals have own phenotypes. Since potential diversity donors are previously selected boars from the past 10 generations, the “oldest” animals are not included in the training set. This is visualized in Fig. 6 with examples of the current generation being in generation 0 or 1. The observed allele frequency in the training population was used as the reference allele frequency for centering in the creation of the genomic relationship matrix used in breeding value estimation. SNP effects for prediction of haplotype effects or GEBVs of old boars were obtained by backsolving, as described on page 195 of Mrode and Pocrnic [58] which refers to Standén and Garrick [59] as the inventors.

Genetic and residual variances were estimated every 3 generations to allow for changing variances due to selection, drift, linkage disequilibrium and the effects of the upcycling pipeline on the elite population. The estimated variances were used for genetic evaluation until they were estimated again. The variance components were estimated with the rrBLUP R package [60] within MoBPS. For computational reasons, only 3000 animals were used for variance component estimation. These were randomly sampled from the current and last 3 generations of the elite population. The observed allele frequencies of these 3000 animals were used as the reference allele frequency to compute the genomic relationship matrix used for variance component estimation. For clarification, different reference allele frequencies were used for variance component estimation, breeding value estimation, and for the relationship matrix used in OCS, reflecting inconsistencies that real breeding programs might have in terms of relationship definitions. Figure 11 in the Appendix shows that different reference allele frequencies result in different estimates of relationship. The motivation to use different genomic relationship estimators for breeding value estimation, variance component estimation and diversity management was to test diversity introduction under practical conditions.

## Results

### Primary results

**Fig. 7:**
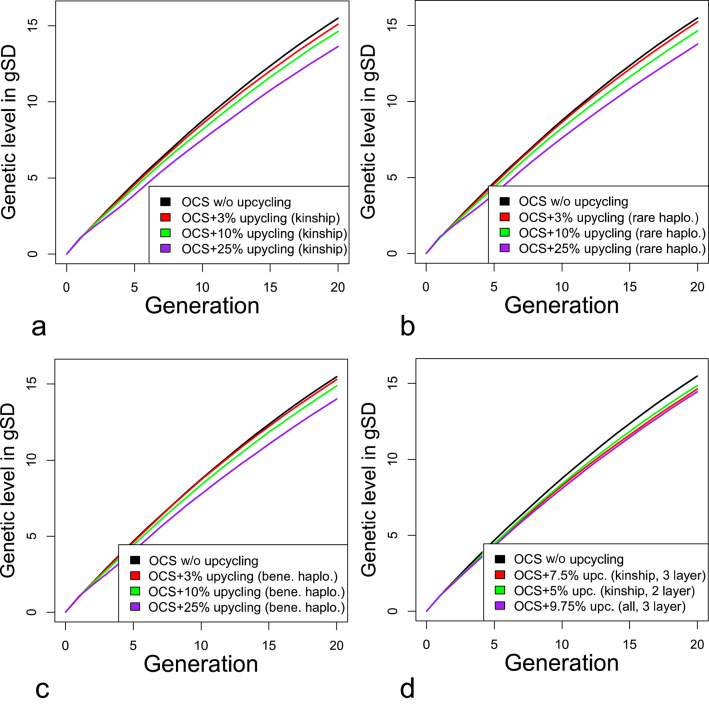
Cumulative genetic gain. The genetic level in the first generation after the burnin period was centered to 0 and the gain is expressed in units of genetic standard deviation calculated as the square root of the genic variance in generation 0. In schemes in which donors are selected based on low kinship to the elite (**a**), carrying haplotypes that are missing in the elite (**b**), carrying haplotypes that are outperforming haplotypes of the elite (**c**), and selected modified schemes (**d**)

Genetic progress was always lower in the multi-layer breeding schemes than in the control scenario (Fig. 7). The difference in genetic progress from the control was larger when more resources were used for the upcycling component. A decline in genetic progress was observed when selecting donors based on low kinship to the elite population. Using 3, 10, and 25% of resources for the upcycling component resulted in genetic levels in the last generation (20) that were 0.26, 0.7 and 1.7 genetic standard deviations (gSD) lower than the control scenario. This is 2.5, 5.5, and 12% lower than the genetic level of the control in generation 20, respectively.

The proportion used for the upcycling component had a higher effect on genetic progress than how diversity donors were selected. For brevity, we will only show results for selection based on low kinship to the elite population in the following. This criterion is simple and appeared to be slightly better for introducing diversity than the others. Results for all other criteria are provided in the Additional File 4 Results S1–S3.

**Fig. 8:**
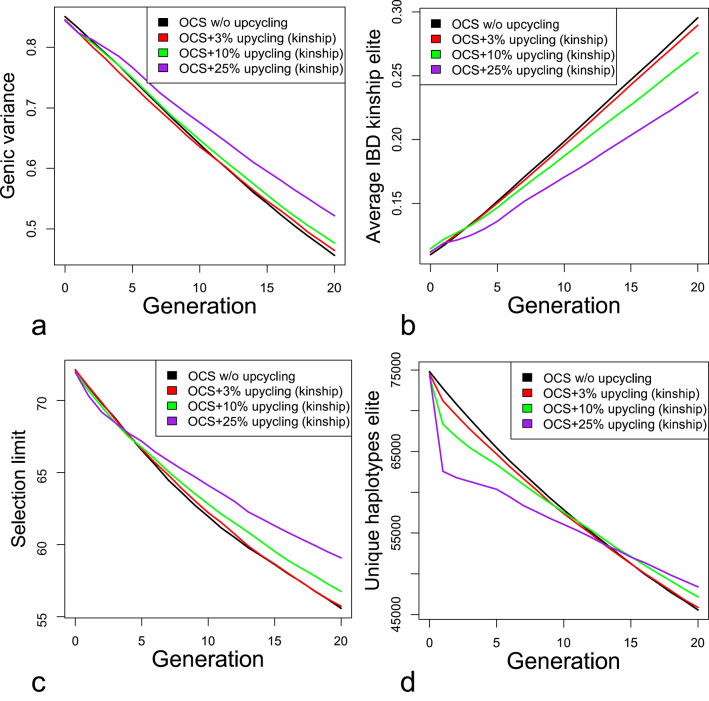
Diversity over time. Development of genic variance (**a**), average kinship (**b**), the selection limit (**c**) and the number of unique haplotypes (**d**) All diversity metrics are measured in the elite population only. The selection limit was calculated as the hypothetical breeding value of the population if all beneficial alleles were fixed. The number of unique haplotypes was calculated by splitting all chromosomes in segments of 1 cM, analyzing the number of unique haplotypes per segment and summing over all segments. Here, it was calculated based on all SNPs, i.e., not only based on SNPs used for genotyping

Diversity in scenarios with and without the upcycling component decreased over time (Fig. 8). However, multi-layer breeding programs lost less diversity per generation. Compared to the level in the last generation of the control scenario, using 25% of resources for upcycling resulted in 0.05 units (or 11% $$\left( {1 - \frac{{geni{c_{control}}}}{{geni{c_{upcycling}}}}*100} \right)$$) higher genic variance, 0.05 lower kinship $$\left( {{f_{control}} - {f_{upcycling}}} \right)$$, a 3.8 gSD higher selection limit, and 1931 or 4% $$ \left( {\frac{{n~haplotypes_{{upcycling}} - n~haplotypes_{{control}} }}{{n~haplotypes_{{control}} }}} \right. $$$$ \left. {*100} \right) $$ more unique haplotypes in the elite population. When the number of unique haplotypes was calculated based on the 20k SNPs used for genotyping, rather than on all approximately 65,000 loci in the genome, about 20k (42%) fewer unique haplotypes were detected in the last generation (not shown), as this subset of SNPs does not tag all truly unique haplotypes. We chose to measure the trait variance as genic variance to remove influences of (past) selection on the genetic variance. Genic variance is calculated as $$\sigma _{{genic}}^{2}=\mathop \sum \limits_{{i=1}}^{{nQTL}} 2{p_i}\left( {1 - {p_i}} \right){{\alpha_i}^2}$$ with $${p}_{i}$$ as the allele frequency of the major allele, and $$a$$ is the additive effect of the beneficial allele.        

Although selection pressure should favor increasing the frequency of the beneficial allele, some beneficial alleles were lost throughout the breeding program (Table 2). Across the 6000 QTL that were simulated, about 5.5% were fixed for the unfavorable allele at the end of the 10 generations burnin period. This implies that about 5670 (94.5%) were either segregating or fixed for the beneficial variant. The more resources were used for diversity introduction, the more beneficial alleles were retained in the elite population after 20 generations.

**Table 2:** Percentage of beneficial alleles that were lost from the elite population over generations in upcycling programs compared to the control program without upcycling

% resources used for upcycling and diversity criterion	Generation 0 (%)	Generation 1 (%)	Generation 5 (%)	Generation 20 (%)
Control without upcycling	5.5	6.3	9.6	18.5
3% kinship	5.5	6.4	9.5	18.1
10% kinship	5.5	6.5	9.4	17.2**
25% kinship	5.5	6.6	9.0	15.5**

Scenario-generation combinations that lost significantly (*p*<0.001) less beneficial alleles than the control scenario are indicated by ‘**’

While multi-layer programs showed greater diversity in the elite population, a fairer comparison to the control scenario would be to express the loss of diversity relative to the genetic gain. Such conversion efficiencies are shown in Table 3 for strategies selecting donors based on low kinship and in Additional File 4 Results S1–S3 for all other criteria. As opposed to the 3 and 10%-kinship scenarios, only the 25%-kinship scenario always showed significantly different efficiencies than the control scenario. In the 25%-kinship scenario, genetic gain per unit gSD lost was 73.7 gSD, a 1% point increase in kinship (f) was converted into 1.1 gSD gain, and a 1 gSD lower selection limit was converted into 0.94 gSD gain. The 25%-kinship scenario only showed lower efficiency with respect to the number of haplotypes, i.e., more haplotypes were lost per unit genetic gain than in the control scenario.

**Table 3:** Efficiencies when selecting diversity donors based on low kinship to elite

% resources used for upcycling and diversity criterion	∆G/∆gSD	∆G/∆f	∆G/∆selection limit	∆G/∆ unique haplotypes (1k)
Control without upcycling	67.8 = 15.7/0.23	0.87 = 15.7/18%	0.87 = 15.7/18.0	0.53 = 15.7/29.3
3% kinship	67.4 = 15.4/0.23	0.88 = 15.4/17%	0.87 = 15.4/17.8	0.53 = 15.4/29.0
10% kinship	68.7 = 15.0/0.22	0.96** = 15.0/16%	0.91 = 15.0/16.5	0.54 = 15.0/27.9
25% kinship	73.7* = 14.0/0.19	1.1** = 14.0/13%	1.0** = 14.0/14.0	0.51** = 14.0/27.2

Efficiencies that are significantly different to the efficiency in the control scenario to the significance level of 1% are indicated with an asterix (*). Significance levels of 0.1% are indicated by ‘**’. Significance was calculated with a paired t-test (function ‘t.test()’ in R). The differences are expressed in absolute differences between generation 0 and generation 20. Differences used in the numerator and denominator of the ratio are provided for reference. These are rounded and thus do not exactly match the presented ratios

Figure 8d shows that the number of unique haplotypes in the elite dropped drastically in the first generation in multi-layer breeding programs. After the initial drop, the number of unique haplotypes decreased at a lower rate than in the control scenario. The initial drop was likely the result of the reduction of elite population size which was due to the reduced number of elite sows. This was confirmed by the observation that the number of unique haplotypes from 100 random elite animals was very similar in early generations between scenarios with different percentages of sows dedicated to the upcycling component (results not shown). This suggests that the number of haplotypes, as defined, is primarily a function of the absolute number of crossovers of a population, which increases with larger population sizes. Similarly, the smaller elite population size may also explain the apparently faster decrease of the selection limit in early generations (Fig. 8c).

The initial faster decrease in haplotype diversity and selection limit (Fig. 8) was interesting since all scenarios were run with OCS in the elite component and they were constrained to the same rate of inbreeding. Also, the observed inbreeding rate was even lower with upcycling than in the control scenario (Fig. 8b), although OCS was restricted to an inbreeding rate of 1% in all cases. Possible explanations are that the genome-wide measure ‘kinship’ is not perfectly indicative of diversity of beneficial alleles or the number of unique haplotypes, or that relationships measured by selected SNPs on the genotyping array do not perfectly indicate the true relationships, as shown in Appendix, Fig. 11.

### Secondary results

The more farrowing pens (dams) were used for the upcycling component, the less were available for elite sows to be inseminated with semen of elite boars. Since the solution space for OCS was restricted to an inbreeding rate of 1% for all scenarios, more boars were selected in schemes with upcycling to compensate for the maternal population size reduction (Table 4). Table 4 also shows that only a small number of boars that were born in the upcycling component were selected as elite sires per generation (1 out of 72 for the 25%-kinship scenario). For all other scenarios, this information is provided in Additional File 4 Results S1–S3.

**Table 4:** Number of boars per origin selected as sires by optimal contribution selection for the elite

% resources used for upcycling and diversity criterion	Layer 1	Layer 2	Layer 3	Layer 4	Elite
Control without upcycling	–	–	–	–	47.29
3% kinship	0	0	0	0.01	49.51
10% kinship	0	0	0	0.05	57.18
25% kinship	0	0.01	0.07	0.92	71.03

Results are averages of the last 5 generations of the simulation. Results are rounded to two decimal positions

On average, about 1, 3, and 6 sows were selected from the upcycling component (layer 1–4) as elite dams based on their GEBV when using 3, 10, and 25% of resources for upcycling, respectively (Table 5). A very limited number of elite sows were selected as dams for the upcycling component based on their low kinship, rather than on GEBV (rightmost column in Table 5). For all other scenarios, this information is provided in Additional File 4 Results S1–S3.

**Table 5:** The number of sows per origin selected based on their GEBV as dams for the elite population, and the number of elite sows selected as dam in the upcycling component based on their diversity

% resources used for upcycling and diversity criterion	Layer 1 for elite	Layer 2 for elite	Layer 3 for elite	Layer 4 for elite	Elite for elite	Elite for diversity
Control without upcycling	–	–	–	–	400	–
3% kinship	0	0.01	0.05	0.84	387.10	0.07
10% kinship	0	0	0.13	2.60	357.28	0.02
25% kinship	0	0	0.36	6.02	293.63	0.07

Results are averages of the last 5 generations of the simulation. Results are rounded to two decimal positions

To investigate whether the lineages of animals selected as elite from the upcycling component were lost from the elite population over time, we took all elite animals in the last generation of the simulation and traced their ancestors back over 10 generations. For the 3, 10, and 25% kinship selection scenarios, 170 out of 2328 (7%), 615 out of 2160 (28%), and 1421 out of 1800 (79%) elite animals had at least 1 diversity donor in their pedigree as an ancestor 10 generations ago or more recent. Based on pedigree analysis, donors had only a very small contribution to the elite population, namely 0.02, 0.1, and 0.4% in the 3, 10, and 25% kinship selection scenarios. This apparent contrast arises because very distant ancestors only have a small contribution. For example, an animal can have potentially 1024 different ancestors 10 generations ago, each one has an expected pedigree contribution of about 0.1% (1/1024 * 100%). The fact that animals with donor-ancestors can be found in the elite shows that descendants of donor x elite matings have a chance of contributing to the elite. However, the low donor-contributions indicate that it takes many generations for descendants of donor x elite matings to arrive in the elite population or to make a meaningful contribution to the elite population. It also indicates that not many donor lineages are stacked, i.e., an elite animal has only a few donor ancestors, if any at all. Overall, the contributed genome fraction of donors to the elite population was very small based on pedigree analysis, and the true contributions may be even smaller.

The diversity donors were typically selected from one of the oldest available generations (Table 6). When selecting based on rare beneficial haplotypes, the selected donors were typically younger, i.e., closer to the current elite animals, compared to donors selected based on low kinship or rare haplotypes.

**Table 6:** Lowest, highest and average generation of selected diversity donors in generation 20

Diversity criterion used for selection	% resources used for upcycling	Oldest generation of selected diversity donors	Average generation of selected diversity donors	Youngest generation of selected diversity donors
Low kinship	3%	10.0	10.3	10.7
	10%	10.0	10.3	11.5
	25%	10.0	10.4	12.2
Rare beneficial haplotypes	3%	10.3	12.1	14.5
	10%	10.1	13.1	17.2
	25%	10.0	13.6	18.5
Rare haplotypes	3%	10.0	10.3	10.8
	10%	10.0	10.5	12.3
	25%	10.0	11.4	15.2
7.5% kinship, 3 layer		10.0	10.3	11.3
5% kinship, 2 layer		10.0	10.3	11.3
9.75% all criteria, 3 layer		10.0	12.1	17.8

The lowest generation donors could be selected from was always 10 generations before the current generation, i.e., generation 10 in generation 20. The only exception is scenario “9.75% all criteria, 3 layer” in which some donors could only be selected 5 generations ago based on carrying rare beneficial haplotypes. Numbers are averages of the 20 replicates per scenario

**Fig. 9:**
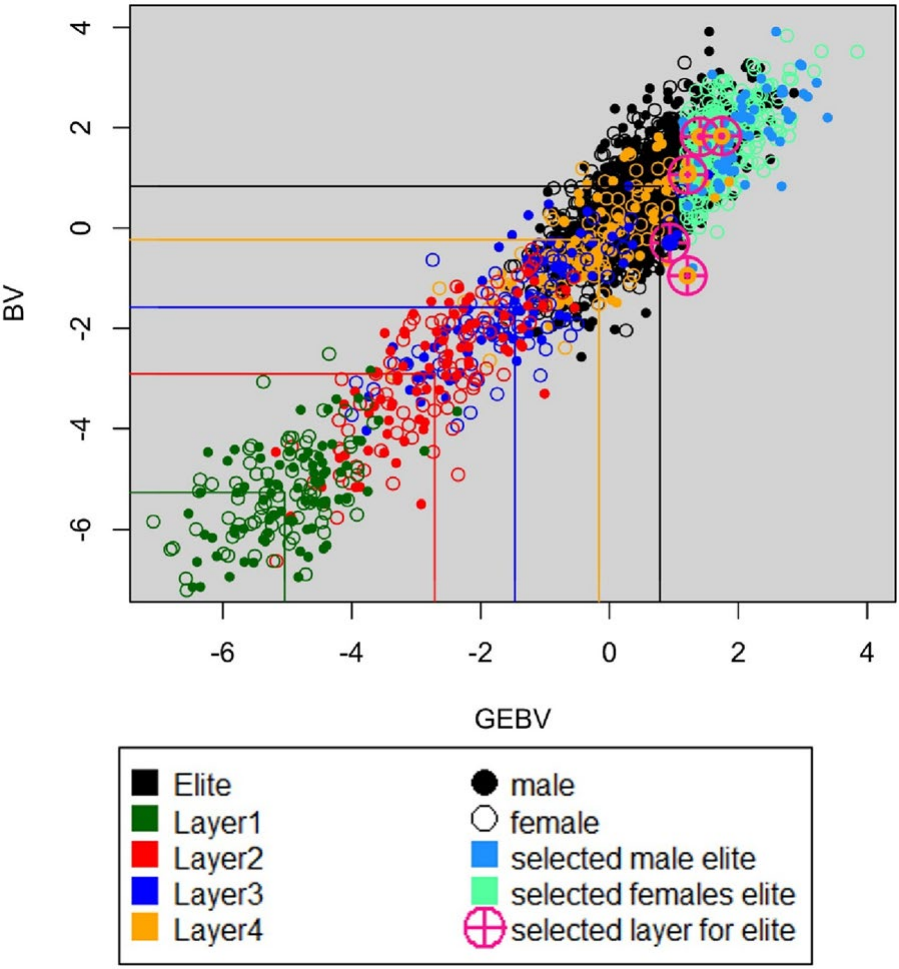
Spread of GEBV and BV in the 25%-kinship scenario. Shown is the last generation of a random replicate. Animals born from Elite x Elite matings are colored black. Females selected based on GEBV as elite parents are colored in light green, and males selected by OCS are colored in bright blue. In this example, four females from layer 4 and one male from layer 3 are selected as elite parents (highlighted by pink crosshairs). Lines indicate the average BV and GEBV for the elite and the upcycling layers

Increasing genetic variance was the most noticeable effect of the upcycling component. This is due to the lower genetic level of donors compared to the elite population. As a consequence, the genetic levels of animals in the upcycling-layers lag behind the one of the elite (Fig. 9). In the 25% kinship scenario, the average breeding value of animals in layers 1, 2, 3 and 4 was 5.5, 3.6, 2.7, and 0.9 gSD lower than the average BV of the elite (gSD here expressed in standard deviation of true breeding values of elite selection candidates). The lag was comparable to that in other scenarios and explains why so relatively few animals were selected from the upcycling component as elite parents (Tables 4 and 5).

**Fig. 10:**
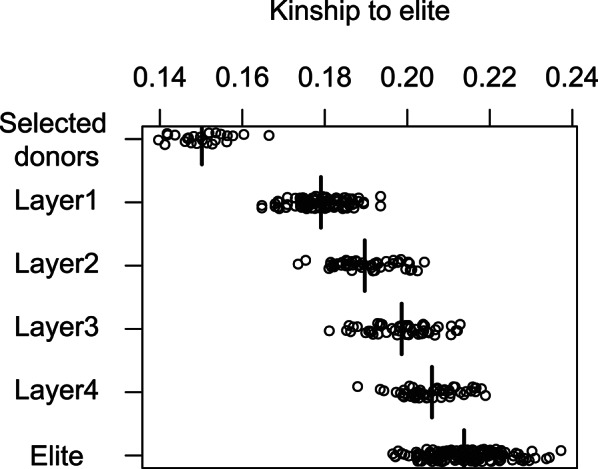
Average IBD kinship to elite animals for animals selected as donors, animals in the upcycling component and elite animals. Shown are values in the last generation from the same replicate of the 25%-kinship scenario as in Fig. 9. Points are jittered to better visualize the distribution. The number of animals is reduced to 50 random animals per layer and 150 random elite animals for better visualization

Apart from increasing the genetic level, the upcycling component also needs to select diverse animals to avoid that backcrossing and selection just reconstructs the genome of the elite population. For reference, the expectation of the average kinship level to the elite after 4 crosses to the elite population, which may be the ancestry of animals born into layer 4 (Fig. 2), was 0.206, based on the average kinship to the elite population of 0.15 for selected donors and 0.21 for elite animals, as shown in Fig. 10 (0.9375*0.21+(1−0.9375)*0.15). Thus, the implemented selection strategy appeared to be successful in keeping a lower kinship to the elite in the upcycling-component than observed for the elite animals (Fig. 10).

## Discussion

The objectives of this study were to design a breeding scheme and test selection criteria to integrate outside genetics into the elite population. The design was evaluated primarily in terms of genetic gain and diversity in an idealized pig breeding scheme without breeding goal changes.

Our results show that introgression of polygenic diversity by means of introduction of old genetics was possible but always compromised genetic gain (Fig. 7). Unless genetic diversity has a value itself, our findings suggest not to introduce outside genetics in commercial populations. However, this may be because of the design of our simulation. Introducing external genetics may be more valuable in practice, depending on the situation. For example, we would expect more benefits of introducing external genetic diversity if (i) the trait definition or the breeding goal changes, as shown by Doekes et al. [28], Leroy et al. [31], and Jacques [32], (ii) if a trait or enough variation for a trait is missing from the elite population e.g. due to a recent critical genetic bottleneck, or (iii) if potential diversity donors show a comparable performance level to the nucleus. The latter may be possible when considering other breeds or lines of competitors (“every pig breeding company has a Pietrain line, and every layer breeder has white layers (Leghorns))”. This was shown by Kinghorn and Kinghorn [61], who considered importation of unrelated but similarly performing animals into an inbred elite population. The multi-layer approach proposed could also be used to merge lines, which may be interesting after acquisition of another breeding program.

Ultimately, the choice of what kind of animals may be considered as diversity donor candidates depends on the goal of the diversity introduction and the availability of sources of diversity. Material with a lower lag in performance can be introduced faster, whereas more genetically distant and, therefore, often lower performing material, may be a source of more variation. We tested three strategies to identify diversity in donor animals, which all performed somewhat similar. This may be because rare (beneficial) haplotypes are mainly discovered in old animals that also happen to be unrelated to the current elite population. The descendants of diversity donors that were selected based on rare (beneficial) haplotypes were not necessarily selected based on the same haplotypes as their donor ancestors. Thus, just selecting the oldest available donors may not perform much worse than an elaborate selection strategy, such as, for example, our selection based on rare beneficial haplotypes.

The three tested indicators to identify diversity donors performed similar in terms of genetic gain and diversity in the elite population, yet selecting based on low average kinship appeared to be the slightly better strategy. This may be because kinship is a genome-wide measure of diversity, as opposed to the other two tested metrics which could result in selecting animals with just a high local diversity (e.g. only some haplotypes are very different but the rest of the genome is already well represented in the elite population). Another reason for the better performance of kinship as a criterion for donor selection may be because of the use of OCS in the elite population. OCS aims to maximize gain for a given rate of increase in kinship. Thus, breeding programs with OCS are expected to perform better when the upcycling component is based on low kinship and not on other diversity metrics.

**Fig. 11:**
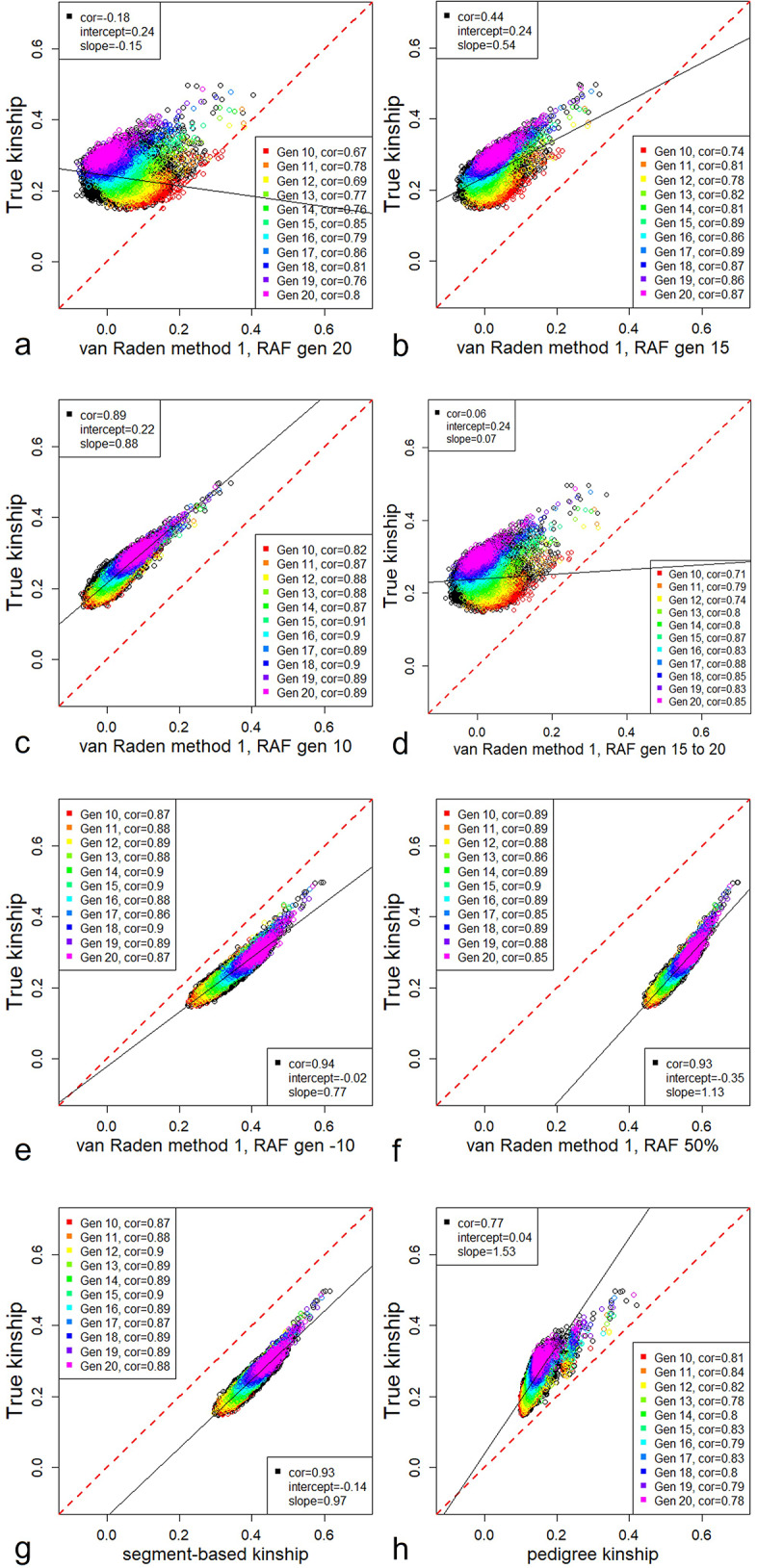
Suitability of kinship estimators. Distribution of different measures of kinships in relation to true IBD kinship obtained by tracing segments back to founders. Only 35 random animals were selected from each generation for better visualization. All values of the off-diagonal elements of kinship matrices are plotted. Plots **a**–**f** show true kinship versus kinship calculated with method 1 of VanRaden [52] using different allele frequencies as reference. The reference allele frequency (RAF) is the observed frequency in generation 20 (**a**), generation 15 (**b**), generation 10 (**c**), generations 15 to 20 (**d**), generation −10 (**e**). The RAF in plot (**f**) is 50% for all SNPs. Plot (**g**) shows the true kinship versus the kinship based on shared identical chromosome segments calculated with function segIBD() of the optiSel R package [51]. Plot (**h**) shows true kinships versus pedigree-based kinships with animals in the first burnin generation (generation −10) as founders. The relationships obtained with method 1 of VanRaden [52] were divided by 2 to convert to kinships. Kinships are calculated for animals in an ongoing genomic selection program. The red dashed line is the ideal regression line with intercept of 0 and slope of 1. Colored points refer to kinships between animals of the same generation

Interestingly, the observed true inbreeding rate in our simulation was lower in scenarios with diversity introduction (Fig. 8b) than in the control scenario, although the inbreeding rate in OCS was constrained to the same amount per generation (1%) in all scenarios. This could be because the 1% restriction is an upper limit, meaning solutions that realize less inbreeding are also possible. However, this was never the case in any replicate. A more plausible explanation may be that the fitted estimated relationship matrix might not capture true relationships well enough (see Appendix, Fig. 11), which means that selection causes more true inbreeding than one would predict. The true inbreeding rate in diversity introduction schemes may be lower because semen from old and, thus somewhat truly unrelated donors is brought into the population. We would expect the same for real breeding programs if semen from the same population is used. The impact on the inbreeding rate when using donors from other populations is difficult to assess, as kinship and thus inbreeding is a measure that expresses diversity relative to founders. However, external donors do not descend from the same founders and are, thus, expected to decrease the IBD-inbreeding rate by definition.

Another reason for the lower inbreeding rates in diversity introduction schemes may be that the genomic prediction training population, which was more diverse when including descendants from donor x elite matings, resulted in more accurate GEBVs for descendants of diversity donors. These animals with a low relationship to the elite population may have more accurate GEBVs as more emphasis is placed on diversity introduction and as the proportion in the training population that is made up of animals with donors in their pedigree increases. This hypothesis is based on Pszczola et al. [62], who reported increasing average reliabilities with decreasing average kinship within the training population. Avendaño et al. [63] found that, when selecting with OCS, contributions are allocated to selection candidates based on “the best available information about their Mendelian sampling terms”, and not their breeding values. For animals with an otherwise low relationship to the elite population, information about the Mendelian sampling terms may be more accurate if they have relatives in the training population and, thus, their estimates are less shrunken. Consequently, families might contribute more evenly to the next generation due to lower variation in within-family accuracy than is anyway the case due to OCS. Note that this is an unproven hypothesis that requires further testing but, if true, it means that the training set design influences the inbreeding rate.

As a reminder, all scenarios were run with the same inbreeding restriction in OCS, which means that the same inbreeding rate is expected in all scenarios. This expectation was not realized, as breeding programs that invested more in diversity introduction showed lower inbreeding rates (Fig. 8b). Thus, breeding programs using diversity introduction may opt to set a less stringent restriction on the inbreeding rate in OCS to still achieve the same true inbreeding rate as in the control scenario and, thereby, accelerate genetic gain.

A weaker restriction of the inbreeding rate in OCS may also result in selecting less boars. Table 4 shows that restructured multi-layer breeding programs select more boars, which we hypothesize is due to the reduced female population size, which had to be compensated by a larger number of boars. In this study, the number of selected boars was not restricted and it was assumed that enough resources were available to house all selected boars. In practice, generations are not discrete as in our simulation, and so it may be that not more boars are selected at a single time point but rather that they are replaced faster. To directly control the number of selected boars, the OCS solutions obtained from optiSel could be changed by fixing the contribution of the lowest-contributing boar to 0 and recalculating optimal contributions. This needs to be repeated until as many boars as desired have positive contributions. Restricting the number of selected boars would disadvantage the genetic gain of breeding programs with diversity introduction more than in programs without diversity introduction.

## Conclusion

Loss of diversity is a threat to livestock populations that we attempted to mitigate by dedicating a portion of the breeding program to introduce new genetics. This simple design was effective in keeping genetic diversity in the population, even when the population was selected with OCS. However, our upcycling scheme resulted in less genetic gain than a conventional breeding program, although it was more efficient in turning the loss of diversity into genetic gain. Effects in real breeding programs may differ to our simulated idealized pig breeding scheme, which was chosen as a benchmark.

## Supplementary Information

Below is the link to the electronic supplementary material.

Additional file 1: Code S1. Calculation, visualization and selection of haplotype estimated breeding values. A small population is simulated with MoBPS for reduced run time to test functionality.

Additional file 2: Code S2. Calculation of unique haplotypes and visualization thereof. The script also shows how we selected based on unique haplotypes. A small population is simulated with MoBPS for reduced run time to test functionality.

Additional file 3: Code S3. Description: Definitions of functions needed for Additional file 1 and Additional file 2.

Additional file 4: Collection of supplementary figures and additional explanation for calculation of unique haplotypes. Results are presented from scenarios in which donors were selected based on rare haplotypes, beneficial haplotypes and selection scenarios that are modified schemes thereof. Figures S1. Toy example for selection based on rare beneficial haplotypes. Figures S2. Small example of calculation of unique identifiers for haplotypes. Results S1. Statistics for selection based on beneficial haplotypes. Results S2. Statistics for selection based on rare haplotypes. Results S3. Statistics for selection in some adjusted scenarios.

## Data Availability

The datasets generated and/or analysed during the current study are not publicly available but are available from the corresponding authors on reasonable request. R scripts to test some of the presented methods are provided in additional files.

## Appendix

The decision to use segment-based kinship is based on preliminary investigations. The following explains our decision process. We ran a control scenario without diversity introduction for 20 generations after 10 generations of burnin. We compared the true identity-by-descent (IBD) kinship, obtained by tracing segments back to founders (animals in generation − 10) with function kinship.emp() of the MoBPS package [54], to various other kinship estimates. The comparisons are shown in Fig. 11. When using method 1 of VanRaden [52], the accuracies of kinships depend hugely on the allele frequency used as reference (RAF) for centering. That differences in choice of RAF result in different genomic relationship estimates was already shown in Table of [52] which shows varying correlations, even negative ones, between pedigree and genomic based inbreeding coefficients depending on the RAF. For intuition, the numerator of the VanRaden [52] relationship matrix for a single locus is $\left({AC}_{animal1}-2RAF\right)\left({AC}_{animal2}-2RAF\right)$, with AC being the allele count at a particular locus (0/1/2). Thus, the numerator is the product of the deviations from the RAF of the two animals. With respect to the diagonal element in a GRM, i.e., the self-relationship, the numerator is the sum over all loci of the square of the deviations of the allele counts of an animal from RAF. Thus, if RAF is the AF of a particular generation, the average relationship within that generation is 0 by design as the average deviation is 0. This can be seen in Fig. 11a for generation 20. For example, using AF of the current generation as RAF would result in estimating higher inbreeding coefficients for ancestors than for current animals. The more ancestral the RAF, the better the kinship estimates of generations that descent from the generation used to obtain RAF (Figs. 11a−c). For us, the ideal kinship estimator does not require the definition of a group of animals to get RAF of a base generation because that base may not unambiguously be known in practice. Using 50% as RAF for all SNPs led to as high correlation to IBD kinships as using RAF of generation − 10 (Figs. 11c, d). Kinships calculated with 50% for RAF reflect the fraction of homozygous SNP markers. Since its slope deviated from 1, we tested the runs-of-identical-sequence based kinship estimator. This kinship was estimated with function segIBD() of the optiSel R package [51]. Using such segment-based estimates resulted in comparable correlation and almost perfect slope, i.e., 1% increase in segment-based-kinship kinship results in about 1% increase in true IBD kinship (Fig. 11e). The pedigree-based kinship is shown in Fig. 11 for completeness too. We did not consider this as a correct and deep pedigree would be needed in practice which may not be available.

Within generation, the correlation between true IBD kinship and different measures was always high (0.67–0.90). However, the accuracy of estimating between-generation kinships varied tremendously. Between-generation kinships are most relevant for selecting donors from past generations for mating to the elite.
