# Supplementary material for: Restructuring breeding programs 1: Integration of diversity
Source: Genet Sel Evol. 2026 Apr 28;58:25. doi: 10.1186/s12711-026-01046-9 (PMC13123036; doi:10.1186/s12711-026-01046-9)
Supplement: Supplementary file 4 — Additional file 4: Collection of supplementary figures and additional explanation for calculation of unique haplotypes. Results are presented from scenarios in which donors were selected based on rare haplotypes, beneficial haplotypes and selection scenarios that are modified schemes thereof. Figures S1. Toy example for selection based on rare beneficial haplotypes. Figures S2. Small example of calculation of unique identifiers for haplotypes. Results S1. Statistics for selection based on beneficial haplotypes. Results S2. Statistics for selection based on rare haplotypes. Results S3. Statistics for selection in some adjusted scenarios. [file 12711_2026_1046_MOESM4_ESM.docx]

| 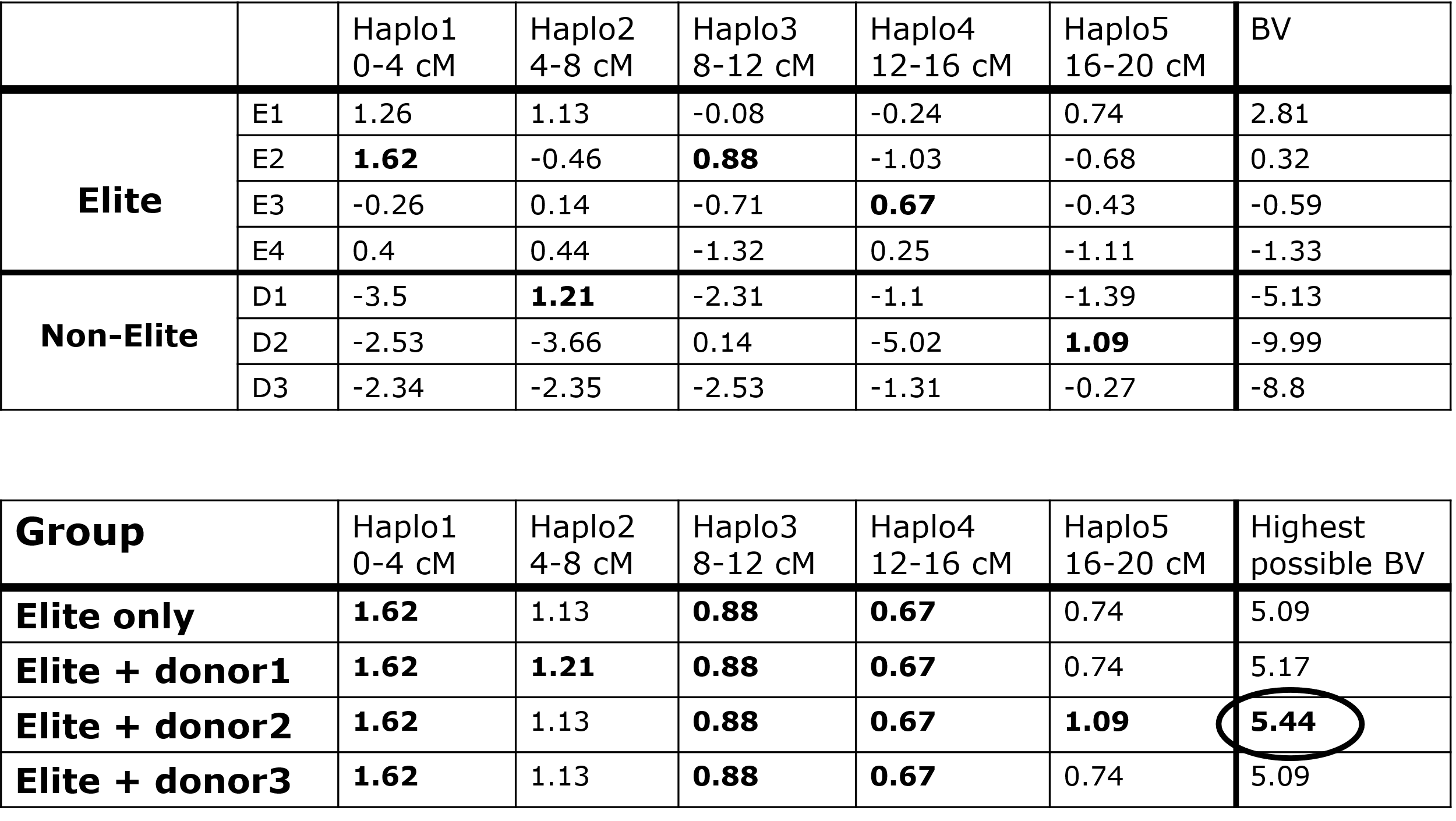 |
| --- |
| Figure S1: Toy example for selection based on rare beneficial haplotypes. Four elite individuals (E1 – E4) and three donor candidates (D1 – D3) are considered in this example. The haplotype with the highest breeding value is indicates in bold. In this example, the second donor would be selected since addition of donor 2 to the elite would increase the highest possible breeding value the most as indicated by the circle. |

To unambiguously assign identifiers to each unique haplotype, the genome is split into segments. The haplotypes are vectors of 1s and 0s. This vector is multiplied with a vector that has as many entries as the number of SNPs in the haplotype and the entry $i$ for the ith SNP has a value of $2^{i}$. Two haplotypes with the same value are thus identical and are labeled with the same haplotype ID. This is visualized in Figure S2.

| 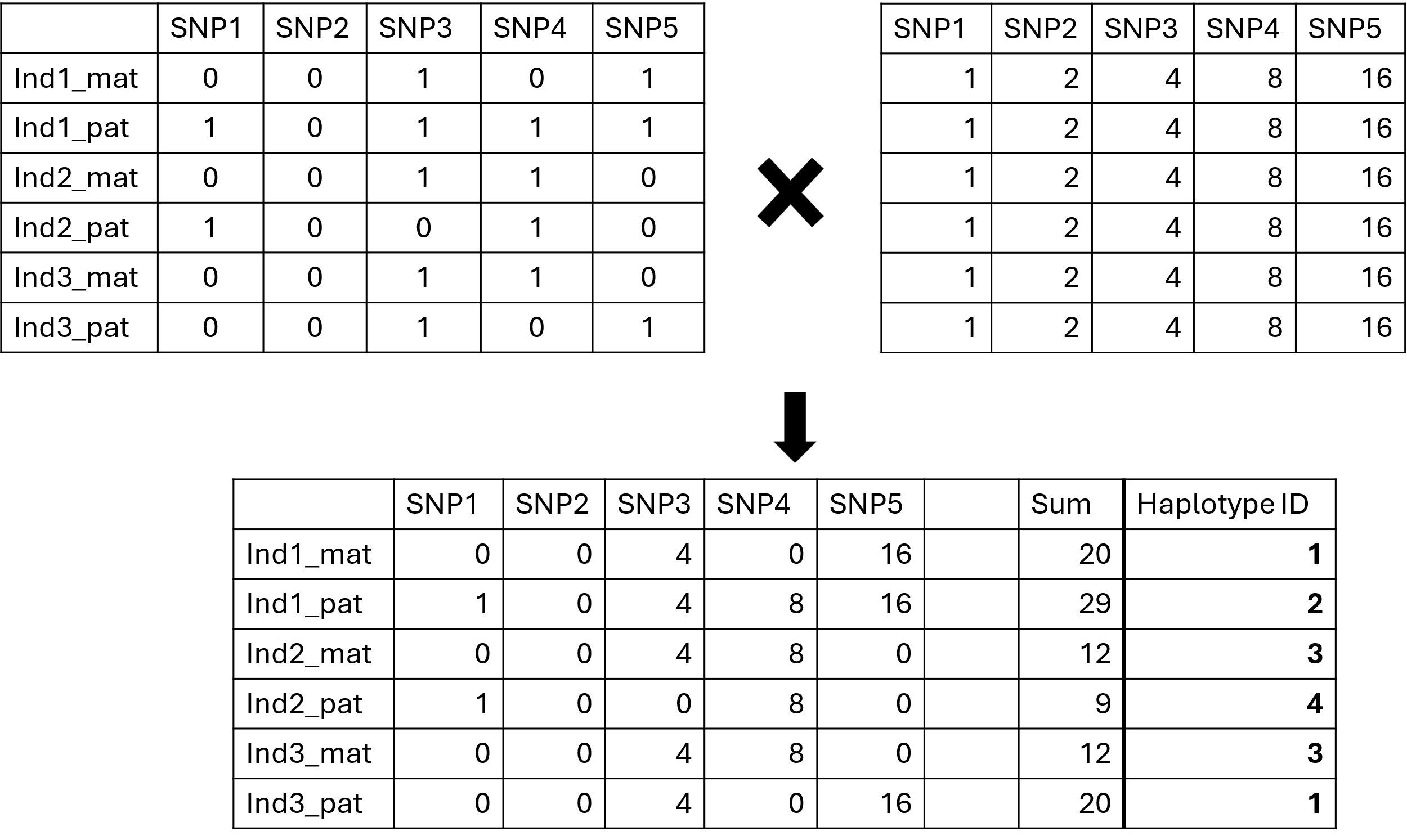 |
| --- |
| Figure S2: Small example of calculation of unique identifiers for haplotypes. |

Results S1: Statistics for selection based on beneficial haplotypes

| 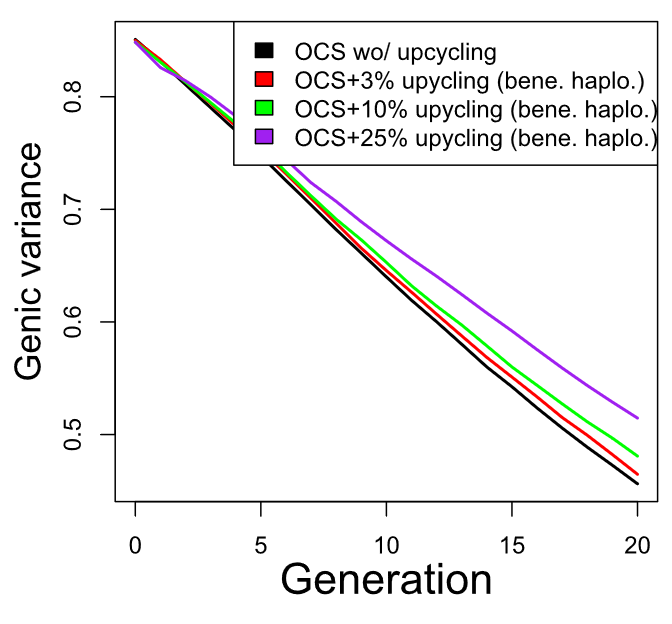  A | 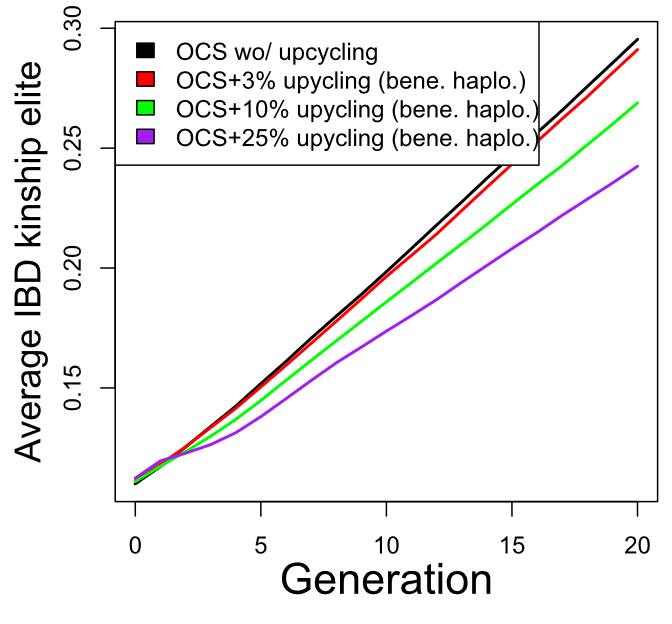  B |
| --- | --- |
| 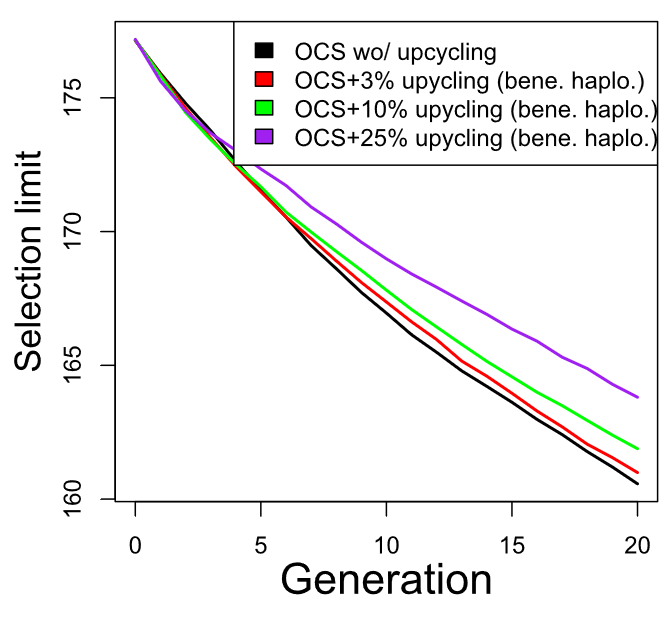  C | 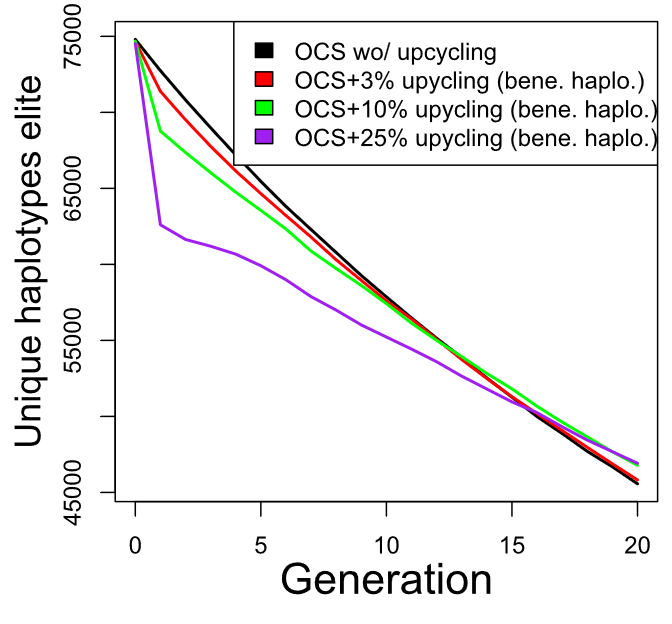  D |
| Development of genic variance (A), average kinship (B), the selection limit (C) and the number of unique haplotypes (D) over time when donors are selected based on carrying missing beneficial haplotypes. All diversity metrics are measured in the elite population only. | |

|  | ∆G/∆SD | ∆G/∆f | ∆G/∆selection limit | ∆G/∆present unique haplotypes (per 1k) |
| --- | --- | --- | --- | --- |
| Control wo/ upcycling | 67.8 =15.7/0.23 | 0.87 =15.7/18% | 0.87 =15.7/18.0 | 0.53 =15.7/29.3 |
| OCS + 3% upcycling (rare beneficial haplo.) | 66.3 =15.5/0.24 | 0.88 =15.5/18% | 0.89 =15.5/17.5 | 0.53 =15.5/29.2 |
| OCS + 10% upcycling (rare beneficial haplo.) | 71.9 =15.3/0.22 | 0.94 ** =15.3/16% | 0.92 =15.3/16.6 | 0.53 =15.3/28.7 |
| OCS + 25% upcycling (rare beneficial haplo.) | 73.4 * =14.4/0.20 | 1.05 ** =14.4/14% | 0.99 * =14.4/14.5 | 0.50 ** =14.4/28.9 |
| Efficiencies that are significantly different to the efficiency in the control scenario to the significance level of 1% are indicated with an asterix (*) Significance levels of 0.01% are indicated by ‘**’. Significance was calculated with a paired t-test (function ‘t.test()’ in R). The differences are expressed in absolute differences between generation 0 and generation 20. Differences used in the nominator and denominator of the ratio are provided for reference. These are rounded thus do not exactly match the presented ratios. | | | | |

|  | Layer 1 | Layer 2 | Layer 3 | Layer 4 | Elite |
| --- | --- | --- | --- | --- | --- |
| Control wo/ upcycling | / | / | / | / | 47.29 |
| OCS + 3% upcycling (rare beneficial haplo.) | 0 | 0 | 0.07 | 0.06 | 49.63 |
| OCS + 10% upcycling (rare beneficial haplo.) | 0.25 | 0.4 | 0.32 | 0.63 | 54.04 |
| OCS + 25% upcycling (rare beneficial haplo.) | 0.18 | 0.34 | 1.16 | 2.28 | 61.64 |
| Number of boars per origin selected by OCS as sires of the next generation when identifying diversity based on rare beneficial haplotypes. | | | | | |

|  | Layer 1 | Layer 2 | Layer 3 | Layer 4 | Elite | Elite to diversity |
| --- | --- | --- | --- | --- | --- | --- |
| Control wo/ upcycling | / | / | / | / | 400 | / |
| OCS + 3% upcycling (rare beneficial haplo.) | 0.02 | 0.02 | 1.37 | 2.73 | 383.88 | 2.32 |
| OCS + 10% upcycling (rare beneficial haplo.) | 0.02 | 0.14 | 3.77 | 7.02 | 349.06 | 6.25 |
| OCS + 25% upcycling (rare beneficial haplo.) | 0.09 | 0.38 | 6.41 | 13.32 | 279.81 | 11.87 |
| Number of sows per origin selected by OCS as sires of the next generation when identifying diversity based on rare beneficial haplotypes. | | | | | |  |

Results S2: Statistics for selection based on rare haplotypes

| 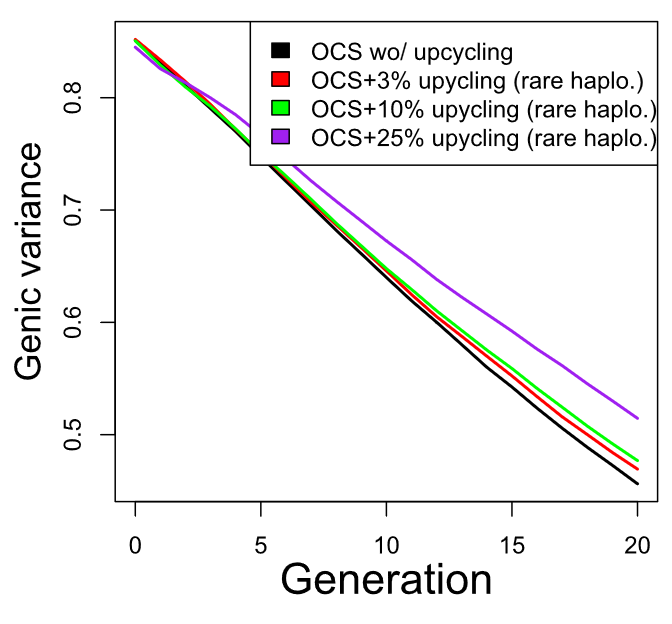  A | 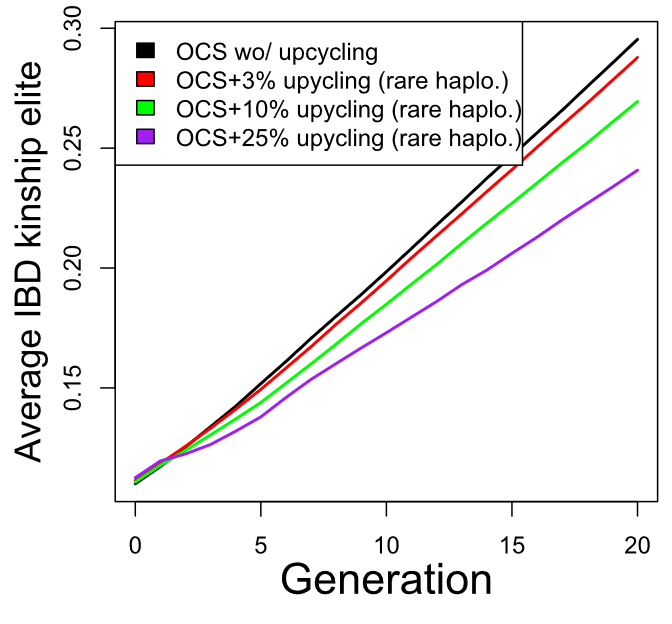  B |
| --- | --- |
| 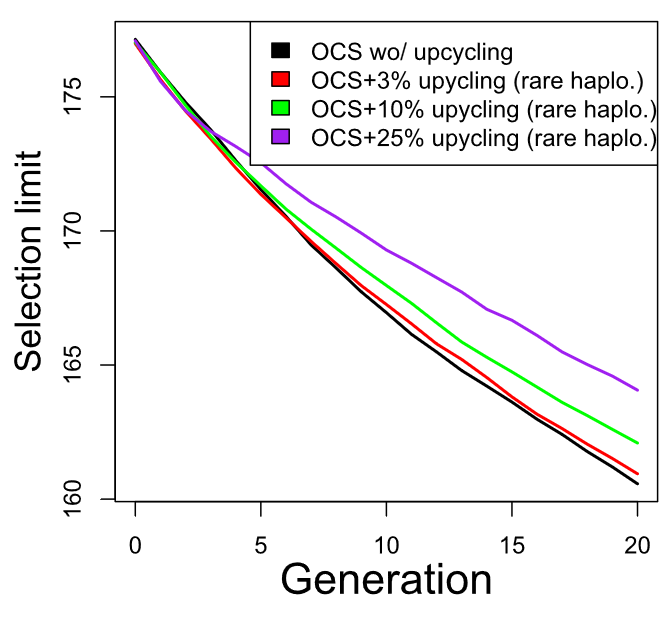  C | 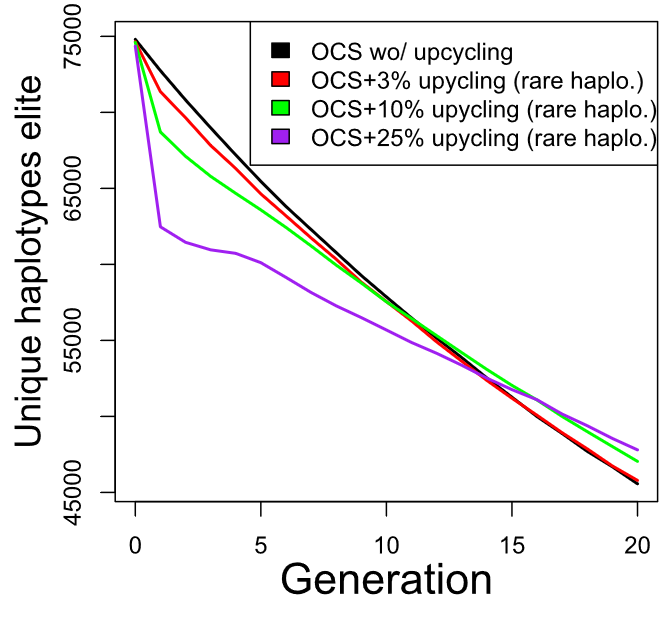  D |
| Development of genic variance (A), average kinship (B), the selection limit (C) and the number of unique haplotypes (D) over time when donors are selected based on carrying missing haplotypes. All diversity metrics are measured in the elite population only. | |

|  | ∆G/∆SD | ∆G/∆f | ∆G/∆selection limit | ∆G/∆present unique haplotypes (per 1k) |
| --- | --- | --- | --- | --- |
| Control wo/ upcycling | 67.8 =15.7/0.23 | 0.88 =15.7/18% | 0.87 =15.7/18.0 | 0.53 =15.7/29.3 |
| OCS + 3% upcycling (rare haplo.) | 65.7 =15.4/0.24 | 0.88 =15.4/18% | 0.89 =15.4/17.5 | 0.53 =15.4/29.3 |
| OCS + 10% upcycling (rare haplo.) | 70.0 =15.0/0.22 | 0.96 ** =15.0/16% | 0.93 =15.0/16.6 | 0.53 =15.0/28.3 |
| OCS + 25% upcycling (rare haplo.) | 74.2 * =14.2/0.19 | 1.10 ** =14.2/13% | 1.0 ** =14.2/14.5 | 0.51 ** =14.2/27.9 |
| Efficiencies that are significantly different to the efficiency in the control scenario to the significance level of 1% are indicated with an asterix (*) Significance levels of 0.01% are indicated by ‘**’. Significance was calculated with a paired t-test (function ‘t.test()’ in R). The differences are expressed in absolute differences between generation 0 and generation 20. Differences used in the nominator and denominator of the ratio are provided for reference. These are rounded thus do not exactly match the presented ratios. | | | | |

|  | Layer 1 | Layer 2 | Layer 3 | Layer 4 | Elite |
| --- | --- | --- | --- | --- | --- |
| Control wo/ upcycling | / | / | / | / | 47.29 |
| OCS + 3% upcycling (rare haplo.) | 0 | 0 | 0 | 0.03 | 48.83 |
| OCS + 10% upcycling (rare haplo.) | 0 | 0 | 0.01 | 0.16 | 55.03 |
| OCS + 25% upcycling (rare haplo.) | 0.01 | 0.08 | 0.15 | 1.34 | 67.54 |
| Number of boars per origin selected by OCS as sires of the next generation when identifying diversity based on rare haplotypes. | | | | | |

|  | Layer 1 | Layer 2 | Layer 3 | Layer 4 | Elite | Elite to diversity |
| --- | --- | --- | --- | --- | --- | --- |
| Control wo/ upcycling | / | / | / | / | 400 | / |
| OCS + 3% upcycling (rare haplo.) | 0 | 0 | 0.13 | 1.56 | 386.32 | 0.41 |
| OCS + 10% upcycling (rare haplo.) | 0 | 0.01 | 0.33 | 4.48 | 355.18 | 0.82 |
| OCS + 25% upcycling (rare haplo.) | 0 | 0.17 | 0.88 | 10.33 | 288.63 | 3.21 |
| Number of sows per origin selected by OCS as sires of the next generation when identifying diversity based on rare haplotypes. | | | | | |  |

Results S3: Statistics for selection in some adjusted scenarios

| 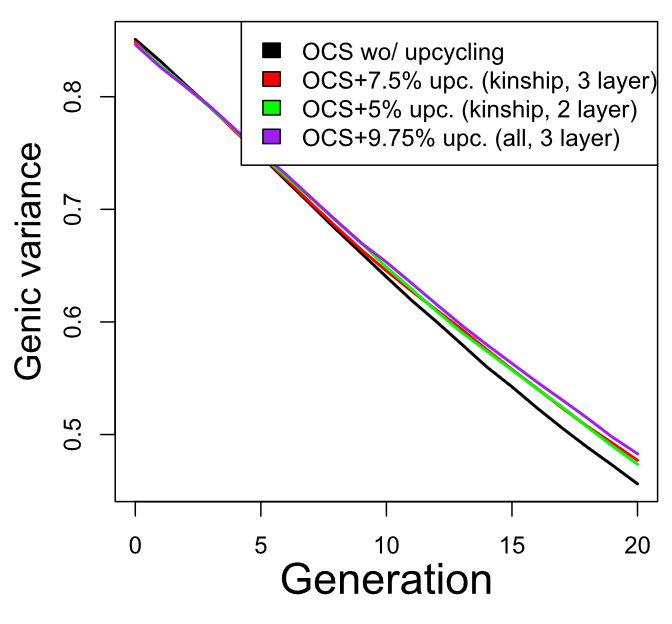 | 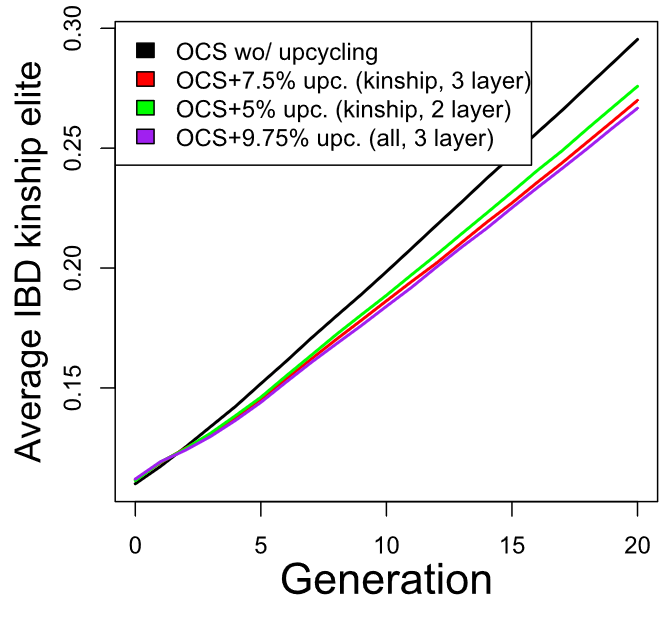 |
| --- | --- |
| 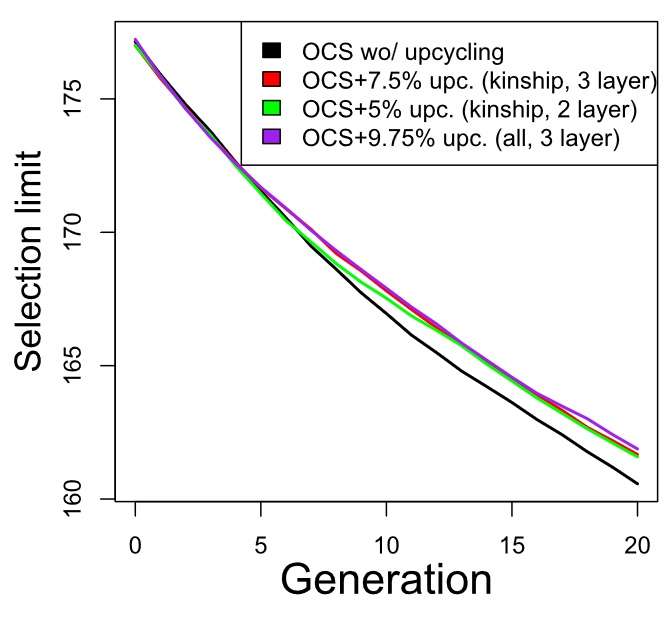 | 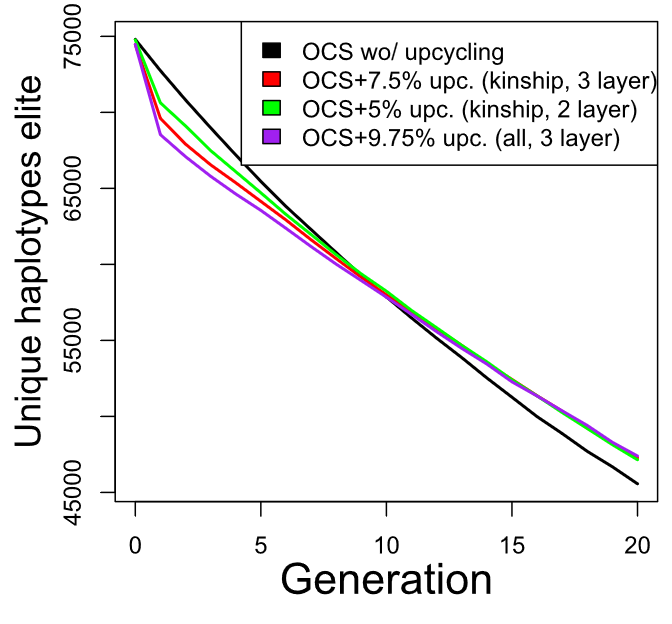 |
| Development of genic variance (A), average kinship (B), the selection limit (C) and the number of unique haplotypes (D) over time in some specially designed schemes. All diversity metrics are measured in the elite population only. | |

|  | ∆G/∆SD | ∆G/∆f | ∆G/∆selection limit | ∆G/∆present unique haplotypes (per 1k) |
| --- | --- | --- | --- | --- |
| Control wo/ upcycling | 67.8 =15.7/0.23 | 0.88 =15.7/18% | 0.87 =15.7/18.0 | 0.53 =15.7/29.3 |
| OCS + 7.5% kinship, 3 layer | 68.8 =15.0/0.22 | 0.96 ** =15.0/16% | 0.90 =15.0/16.6 | 0.54 =15.0/27.7 |
| OCS + 5% kinship, 2 layer | 70.04 =15.1/0.22 | 0.95 ** =15.1/16% | 0.90 =15.1/16.7 | 0.55 * =15.1/27.3 |
| OCS + 9.75% all criteria, 3 layer | 69.1 =14.8/0.22 | 1.00 ** =14.8/15% | 0.89  =14.8/16.7 | 0.55 * =14.8/26.9 |
| Efficiencies that are significantly different to the efficiency in the control scenario to the significance level of 1% are indicated with an asterix (*) Significance levels of 0.01% are indicated by ‘**’. Significance was calculated with a paired t-test (function ‘t.test()’ in R). The differences are expressed in absolute differences between generation 0 and generation 20. Differences used in the nominator and denominator of the ratio are provided for reference. These are rounded thus do not exactly match the presented ratios. | | | | |

|  | Layer 1 | Layer 2 | Layer 3 | Layer 4 | Elite |
| --- | --- | --- | --- | --- | --- |
| Control wo/ upcycling | / | / | / | / | 47.29 |
| OCS + 7.5% kinship, 3 layer | 0 | 0 | 0.03 | / | 57.98 |
| OCS + 5% kinship, 2 layer | 0 | 0 | / | / | 56.83 |
| OCS + 9.75% all criteria, 3 layer | 0.02 | 0.18 | 0.27 | / | 59.43 |
| Number of boars per origin selected by OCS as sires of the next generation in some adjusted scenarios. | | | | | |

|  | Layer 1 | Layer 2 | Layer 3 | Layer 4 | Elite | Elite to diversity |
| --- | --- | --- | --- | --- | --- | --- |
| Control wo/ upcycling | / | / | / | / | 400 | / |
| OCS + 7.5% kinship, 3 layer | 0 | 0 | 0.65 | / | 369.35 | 0 |
| OCS + 5% kinship, 2 layer | 0 | 0.01 | / | / | 379.99 | 0 |
| OCS + 9.75% all criteria, 3 layer | 0.01 | 0.76 | 2.83 | / | 357.40 | 1.39 |
| Number of sows per origin selected by OCS as sires of the next generation in some adjusted scenarios. | | | | | |  |
